# Supplementary material for: Fabrication of Encapsulated Gemini Surfactants
Source: Molecules. 2022 Oct 7;27(19):6664. doi: 10.3390/molecules27196664 (PMC9573393; doi:10.3390/molecules27196664)
Supplement: Supplementary file 1 [file molecules-27-06664-s001.zip › molecules-1879563-supplementary.pdf]

SUPPLEMENTARY MATERIALS

Fabrication of encapsulated gemini surfactants

Bogumił Brycki *, Adrianna Szulc and Iwona Kowalczyk

Synthetic details:

In all gemini surfactants synthesis, 1 equivalent of the appropriate diamine (TMPDA for 10(E)-3-10(E) Cl and 12-3-12; BDMAEE for 10(E)-O-10(E) Cl, 12-O-12, 12(E)-O-12(E) Cl and 12(E)-O-12(E) Br) was mixed with 2 equivalents of halogenoester (10Cl for 10(E)-3-10(E) Cl and 10(E)-O-10(E) Cl; 12Cl for 12(E)-O-12(E) Cl; 12Br for 12(E)-O-12(E) Br), or bromododecane for 12-3-12 and 12-O-12. The reactions were carried out without a solvent, at room temperature, by stirring using a magnetic stirrer until the reaction mixture solidified (max 6 h). The crude products were crystallized from a mixture of acetoni-trile:methanol in the volume ratio of 10:1 and dried in an incubator (60 °C) and over P4O10 in a vacuum desiccator.

10(E)-3-10(E) Cl: *N,N,N′,N′*-tetramethyl-1,3-propanediamine (TMPDA) (0.1 mol; 13.02 g) was placed in a 250 ml round-bottom flask and decyl chloroacetate (10Cl) (0.2mol; 46.9 g) was added. Reaction was carried out without the solvent, at room temperature, with stirring using a magnetic stirrer, until the reaction mixture solidified. After completion of the reaction, the crude product was purified by crystallization in acetonitrile/methanol (10:1). The product was filtered and dried in an incubator (60°C) and over P_4O_{10} in the vacuum desiccator. The yield of the reaction was >85%.

10(E)-O-10(E) Cl: bis[2-(*N,N*-dimethylamino)ethyl] ether (BDMAEE) (0.1 mol; 16.03 g) was placed in a 250 ml round-bottom flask and decyl chloroacetate (10Cl) (0.2 mol; 46.9 g) was added. Reaction was carried out without the solvent, at room temperature, with stirring using a magnetic stirrer, until the reaction mixture solidified. After completion of the reaction, the crude product was purified by crystallization in acetonitrile/methanol (10:1). The product was filtered and dried in an incubator (60°C) and over P_4O_{10} in the vacuum desiccator. The yield of the reaction was >90%.

12-3-12: *N,N,N′,N′*-tetramethyl-1,3-propanediamine (TMPDA) (0.1 mol; 13.02 g) was placed in a 250 ml round-bottom flask and 1-bromododecane (0.2 mol; 49.8 g) was added. Reaction was carried out without the solvent, at room temperature, with stirring using a magnetic stirrer, until the reaction mixture solidified. After completion of the reaction, the crude product was purified by crystallization in acetonitrile/methanol (10:1). The product was filtered and dried in an incubator (60°C) and over P_4O_{10} in the vacuum desiccator. The yield of the reaction was >95%.

12-O-12: bis[2-(*N,N*-dimethylamino)ethyl] ether (BDMAEE) (0.1 mol; 16.03 g) was placed in a 250 ml round-bottom flask and 1-bromododecane (0.2 mol; 49.8 g) was added. Reaction was

carried out without the solvent, at room temperature, with stirring using a magnetic stirrer, until the reaction mixture solidified. After completion of the reaction, the crude product was purified by crystallization in acetonitrile/methanol (10:1). The product was filtered and dried in an incubator (60°C) and over P_4O_{10} in the vacuum desiccator. The yield of the reaction was >95%.

12(E)-O-12(E) Cl: bis[2-(*N,N*-dimethylamino)ethyl] ether (BDMAEE) (0.1 mol; 16.03 g) was placed in a 250 ml round-bottom flask and dodecyl chloroacetate (12Cl) (0.2 mol; 52.5 g) was added. Reaction was carried out without the solvent, at room temperature, with stirring using a magnetic stirrer, until the reaction mixture solidified. After completion of the reaction, the crude product was purified by crystallization in acetonitrile/methanol (10:1). The product was filtered and dried in an incubator (60°C) and over P_4O_{10} in the vacuum desiccator. The yield of the reaction was >85%.

12(E)-O-12(E) Br: bis[2-(*N,N*-dimethylamino)ethyl] ether (BDMAEE) (0.1 mol; 16.03 g) was placed in a 250 ml round-bottom flask and dodecyl chloroacetate (12Br) (0.2 mol; 61.4 g) was added. Reaction was carried out without the solvent, at room temperature, with stirring using a magnetic stirrer, until the reaction mixture solidified. After completion of the reaction, the crude product was purified by crystallization in acetonitrile/methanol (10:1). The product was filtered and dried in an incubator (60°C) and over P_4O_{10} in the vacuum desiccator. The yield of the reaction was >85%.

Elemental analysis:

surfactant	found			calculated		
	%C	%H	%N	%C	%H	%N
10(E)-3-10(E) Cl	61.91	10.64	4.59	62.08	10.76	4.67
10(E)-O-10(E) Cl	60.89	10.23	4.38	61.03	10.56	4.45
12-3-12	58.81	10.56	4.17	59.22	10.90	4.46
12-O-12	58.48	11.19	4.15	58.35	10.71	4.25
12(E)-O-12(E) Cl	62.91	10.98	4.03	63.04	10.88	4.08
12(E)-O-12(E) Br	55.96	10.02	3.59	55.81	9.63	3.62
